# Obstetric and psychological characteristics of women seeking multiple abortions in the region of Monastir (Tunisia): results of a cross-sectional design

**DOI:** 10.1186/s12905-015-0198-x

**Published:** 2015-05-10

**Authors:** Sana El Mhamdi, Arwa Ben Salah, Ines Bouanene, Imen Hlaiem, Saloua Hadhri, Wahiba Maatouk, Mohamed Soltani

**Affiliations:** Department of Community Medicine, Faculty of Medicine of Monastir, Monastir, Tunisia; Reproductive Health Center of Monastir, Monastir, Tunisia

**Keywords:** Abortion, Induced, Aged, Contraception, Health Knowledge, Attitudes, Practice, Violence, Mental disorders

## Abstract

**Background:**

Repeat abortion is a public health concern favored by many obstetric and social factors.

The purpose of our study was to identify associated factors to repeated abortion in the region of Monastir (Tunisia). Common mental disorders (CMD) such as anxiety and depression were also evaluated in women seeking voluntary repeated abortion.

**Methods:**

We carried out a cross sectional study between January and April 2013 in the Reproductive Health Center (RHC) of the region of Monastir in Tunisia (This study is part of a prospective design on mental disorders and intimate partner violence among women seeking abortions in the RHC).

Among women referred to the RHC we selected those seeking voluntary abortion (medical or surgical method). Data on women’s demographic characters, knowledge and practices about contraceptive methods and abortion were collected the abortion day via a structured questionnaire. Data about anxiety and depression status were evaluated during the post-abortal control visit at 3–4 weeks following pregnancy termination.

**Results:**

Of the 500 interviewed women, 211 (42.2 %; CI_95%_ [37.88 – 46.52]) were seeking repeated abortions. Multivariate analysis showed that increased age, lower level of women school education, single status, poor knowledge about birth control methods and history of conflict/abuse by a male partner, were uniquely associated with undergoing repeat compared with initial abortion.

CMD were significantly higher in women undergoing second or subsequent abortion (51.1 %) single and lower educated women. Women relating a history of conflicts/abuse report more CMD than others (30.6 % vs 20.8 %).

**Conclusion:**

Health facilities providing abortion services need to pay more attention to women seeking repeat abortion. Further studies are needed to well establish the relation between the number of abortion and the occurrence and the severity of CMD.

## Background

Abortion is one of the most commonly performed procedures in the world. In 2003, about 41.6 million induced abortions were performed worldwide, and that number has remained fairly constant over the last 10 years [1].

The incidence of women seeking induced abortion and especially those seeking repeated induced abortion is an important indicator of the frequency with which women experience unintended pregnancies, and it can point to gaps in contraceptive services and effective contraceptive use [2].

Repeated induced abortion seems to be a public health concern in many countries especially after liberalization of abortion laws [3]. This practice can lead to many sexual, reproductive and psychological health problems [4].

Previous research, mainly done in high-income countries, showed that repeated abortion is common in women with higher age, higher parity, and lower socioeconomic status [3]. However, there is no stronger evidence to suggest that women seeking repeat abortion are using it as a birth control method. Evidence also does not indicate that women seeking repeat abortion have mental illnesses [5]. But, it is actually recognized that women who had abortions were more likely than delivering women to develop common mental disorders (CMD), as anxiety, depression substances abuse [6].

Despite the high incidence of repeat abortions and theirs consequences, researches on it are scarce in low and middle-income countries.

In Tunisia the legalization of abortions was done since 1973 and the contraceptive coverage reached 60.2 % in 2006. The abortion rate among childbearing age women was about 7.8 % in 2009 [7]. However, data on repeated abortion and the profile of women seeking it are not available as other low and middle-income countries. Information’s about women’s mental issues after abortion were also not available.

Available data on repeated abortion can be helpful in understanding the social significance of this practice and informing policies for sexual and reproductive health.

The aim of our study was to identify associated factors to repeated abortion in the region of Monastir (Tunisia). CMD (anxiety and depression) were also evaluated in women seeking voluntary repeated abortion.

## Methods

### Setting and sampling

A cross-sectional survey was carried out between January and April 2013 in the reproductive health center (RHC) of the region of Monastir (Tunisia). The study is part of a prospective study on mental disorders and intimate partner violence among women seeking abortions in the RHC (Monastir, Tunisia). The RHC ensures most of medical and surgical abortions in the Monastir’s region. In few cases abortions could be performed in the private sector.

RHC provided activities of pregnancies follow-up, family planning, management of sexually transmitted infections and medical/surgical abortion.

Among women referred to the RHC we selected those seeking voluntary abortion (medical or surgical method). We excluded women with an ectopic pregnancy, molar pregnancy, ended pregnancy and women looking for therapeutic abortion.

The minimum sample size required for the study was 372 women based on 0.05 probability of a type 1 error (a), a precision of 2 % and assuming rate of repeated voluntary abortion about 41 % according to literature [8].

### Measures

We conducted a simple random sampling. A questionnaire including women’s demographic characters, knowledge and practices about contraceptive methods and abortion was used. Data were collected the abortion day using a face to face interview.

After sampling procedure, data were collected via a structured questionnaire developed this instrument on the basis of the research literature [9] and including the following items:

- Women’s demographic and obstetric character
- Women’s knowledge and practices about contraceptive methods
- Women’s knowledge and practices about abortions
- CMD during the post-abortal visit (3 – 4 weeks after abortion)

Anxiety and depression were evaluated using the Hospital Anxiety and Depression scale (HADS). Depressive and anxiety symptoms are defined by the HADS questionnaire as: none (score 0–6); depressive mood/mild or moderate anxiety (score 7–10); and risk for depression/possible anxiety disorder (score >10). Two trained investigators interviewed each respondent.

### Data analysis

Data analysis was carried out in SPSS version 20.0 and p-values < 0.05 were considered statistically significant. Women were divided in two categories (first abortion and repeated abortion). Anxiety and depressive scores on the HADS were categorised as none (score ≤ 10) or present (score > 10), to clarify whether anxiety and depression symptoms were present or not.

Univariate analysis was performed using the Chi-Square test. The relationship between CMD and abortive episodes was also evaluated using the Chi-Square test.

A multivariate stepwise logistic regression model was used to identify factors associated with repeated abortions. In this case, a woman seeking repeated abortion was our dependant variable. Explicative variables with a univariate test value ≤ 0.25 were included. The final retuned variables were those significant at the level of 5 %.

### Ethical consideration

In our protocol we explained that no woman will be obliged to participate in this study. In fact, for each woman we explained the aim of the study, that participation and data provided will be completely confidential. Their name will not be used in any report of the study. Given that we have taken inti account all these precaution, the “ethical committee of the University Hospital of Monastir” approved our study with only the verbal consent before the women’s interview.

## Results

A total of 500 women seeking abortions in the RHC were interviewed from them 211 (42.2 %; 95 %CI [37.88 – 46.52]) seeking repeated abortions. Of the 211 women seeking repeated abortions, 126 (59.7 %) were undergoing a second voluntary abortion, 65 (30.8 %) a third abortion, and 20 (9.5 %) a fourth or subsequent abortion.

Women undergoing repeat abortion (mean age 32 ± 6 years) were significantly older than those undergoing abortion for the first time (means age 30.1 ± 5 years).

Women seeking first abortion were more likely to attend higher level of school education than women seeking repeat abortion (20.4 % vs 10.4 %). Partner of women seeking first abortion were also more likely to attend higher level of school education (Table 1).

**Table 1:** Baseline and obstetric characteristics of women who seeks abortion of the first time and repeater

Characteristic	First abortion; n (%)	Repeat abortion; n (%)	p
**Age (years)**			< 0.001
<20	6 (2)	5 (2.5)	
20 – 35	238 (82.4)	135 (63.9)	
>35	45 (15.6)	71 (33.6)	
**Area**			0.11
Urban	236 (91)	184 (87.2)	
Rural	26 (9)	27 (12.8)	
**Education**			<0.001
Less than high school	104 (36)	106 (50.2)	
High school diploma	126 (43.6)	83 (39.3)	
More than high school	59 (20.4)	22 (10.4)	
**Household income**			<0.001
Economic problems	73 (25.3)	77 (36.6)	
**Marital status**			<0.001
Single	28 (9.7)	50 (23.7)	
Married	256 (88.5)	154 (72.9)	
Cohabitation	5 (1.8)	7 (3.4)	
**Number of children**			0.016
One or more	252 (87.2)	168 (79.6)	
**Knowledge about contraceptive methods**			<0.001
Yes	152 (52.6)	56 (26.5)	
**Birth control method**			<0.001
None	198 (65.4)	166 (78.7)	
Oral contraceptive pills user	72 (24.9)	36 (17.1)	
Intrauterine device user	3 (1)	1 (0.5)	
Condom user	14 (4.8)	6 (2.8)	
Others	2 (3.9)	2 (0.9)	
**Partner education**			<0.001
Less than high school	108 (37.4)	102 (48.3)	
High school diploma	125 (43.3)	91 (43.1)	
More than high school	56 (19.4)	18 (8.5)	
**History of conflicts/physical or sexual abuse**	40 (13.9)	90 (42.8)	<0.001
**Common mental disorders**	69 (23.9)	66 (31.3)	0.041

The rate of single women was also significantly higher in women seeking repeat abortion (23.7 % vs 9.7 % in first abortion women).

Women presenting for second or subsequent abortion report significantly more conflicts with their partner, physical or sexual violence (Table 1).

Women presenting for repeat abortion were less likely than those presenting for a first abortion to have adequate knowledge about birth control methods and a less use of these methods at some point (*p* < 0.001).

Characteristics examined in stepwise multivariate logistic regression analysis including age, women and partner education, marital status, knowledge and use of birth control methods, area, household income and history of conflicts/abuse indicated that increased age, lower level of women school education, single status, poor knowledge about birth control methods and history of conflict/abuse by a male partner, were uniquely associated with undergoing repeat compared with initial abortion (Table 2).

**Table 2:** Factors associated with repeat abortion. Multivariate logistic regression analysis (N = 500)

Variable	OR_a_	95 % CI	p
Age (years)			0.002
20 – 34	1	-	
<20	1.4	1.13 – 1.73	
>35	2.1	1.36 – 3.23	
Marital status			<0.001
Married	1	-	
Cohabitation	2.33	1.41 – 3.86	
Single	4.87	2.78 – 8.55	
Level of education			<0.001
More than high school	1	-	
High school diploma	1.64	1.25 – 2.15	
Less than high school	2.96	1.65 – 5.32	
Knowledge about contraceptive methods			<0.001
Yes	1	-	
No	2.61	1.69 – 4.04	
History of conflicts/abuse			<0.001
No	1		
Yes	1.98	1.36 – 2.88	

^a^adjusted

The study of the relationship between CMD and women characteristics showed that women undergoing second or subsequent abortion were more likely to develop CMD (31.3 % vs 23.9 %). The rate of CMD was significantly higher in single than married women and in lower educated women (Table 3). Finally, women relating a history of conflicts/abuse were more likely to develop CMD than others (30.6 % vs 20.8 %) (Table 3).

**Table 3:** Common Mental Disorders according to women baseline and obstetric characteristics

Variable	Common mental disorders; n (%)	p
**Abortion**		0.041
First abortion	69 (23.9)	
Repeat abortion	66 (31.3)	
**Age (years)**		0.31
≥35	102 (27.7)	
>35	33 (26.8)	
**Marital status**		0.048
Married	90 (21.6)	
Cohabitation	3 (27.6)	
Single	33 (44.4)	
**Education**		0.008
Less than high school	70 (33.3)	
High school diploma	52 (24.9)	
More than high school	13 (16)	
**History of conflicts/abuse**		0.032
Yes	40 (30.6)	
No	77 (20.8)	

## Discussion

The aim of our study was to identify the rate of women seeking repeated abortions in the region of Monastir (Tunisia) and associated factors. The relationship between CMD and women characteristics was also investigated.

In Tunisia, voluntary abortion is a public health issue. In fact, since the legalization of the abortion low (1973), the number of abortions in the public sector had increased from 342 in 1973 to 14699 in 2009 [4]. Data about the profile of women seeking repeat abortion are scarce. Data on psychological impact of abortion were also scarce.

Our results found that 42.2 % of women seeking abortion had experienced one, two or subsequent abortions. This rate is similar to those noted in developed countries such as Sweden [8], USA [10] and England [11].

Repeat abortion was associated with some baseline social and obstetrical factors. Our cross-sectional study identifies 5 characteristics that are independently correlated to repeat abortion; some of them are in agreement and others are in contradiction with previous studies.

We found an association between repeat induced abortion and increased age. Compared with first-time abortion patients, repeat-abortion patients were significantly older than those, like in the USA and Canada [9, 10]. Aged women are often those who have a sufficient number of children so they undergo an abortion in case of unintended pregnancy. There is marked shift in age of multiple time abortion seekers; the most relevant age-group is 15–29 years in previous studies [12]. The fact that women seeking one abortion are younger than women seeking multiple abortions confirms that women undergoing an abortion are at high risk of repeat unintended pregnancy [13]. Thus, interventions that improve women’s knowledge and practice in order to better manage their lives and reduce induced abortions are needed.

Recurrent abortion seekers were less educated than first-time abortion seekers, and higher per cent of them were single. It suggests that repeaters have less stable family relations compared to the first time abortion seekers, which is in accordance with previous studies [8, 14, 15]. In a Danish study, single women were more likely to seek repeat abortions compared to first abortions ones (ORa = 39.1) [16]. The same facts were reported in Scotland and Filand [17, 18].

Repeat abortions seem to be closely related to the lack of knowledge about birth control methods in our study. Our results were also in accordance with results of studies in many developed and developing countries [19, 20]. Consultations providing information’s about contraception methods and their availability free of charge especially for lower educated women are needed. It is not a matter of use rather than a problem of knowledge. Women did not know how to deal with missing a pill and had very little awareness of forms of contraception and emergency contraception [21, 22]. According to Serrano et al. [23] failure of contraceptive use was reported by 77 % of those using condoms and by 84 % of those using hormonal contraception. In fact, a rise in contraceptive use must be combined to effectiveness of use to obtain a decline in induced abortion.

Actually, it is well recognized that induced abortion was associated with CMD mainly anxiety and depression during the post-abortal period [24, 25]. Our findings suggested a relationship between repeat abortion and history of conflicts or violence (physical/sexual abuse by a male partner) suggest serious effects of these factors on women's health outcomes [26]. In fact, these women were more likely to develop CMD as suggested by our results and others studies. History of conflicts, physical and sexual abuse was closely related with social problems, request for a termination of pregnancy and a higher risk of anxiety, depression and sleep disturbances [27].

According to literature, a relationship exists between pregnancy loss and CMD (anxiety and depression) and drug use during the subsequent year [28, 29]. Giannandrea et al. [29] showed that pregnancy loss type was not related to depression, although the losses’ number was related to the presence of depression and anxiety.

### Limitations

The main limitation of our study is its cross sectional design carried out over a short period. In this case we cannot draw conclusions about a causal relationship between repeat abortions and the occurrence of CMD. Nevertheless, CMD and partner violence have not been studied yet in women seeking abortions in Tunisia. So, this study can be a first step in assessing these aspects. Another limitation was the use of women seeking first abortion as a control group which does not allow us the comparison to women without a history of abortion. However, the estimation of CMD in women seeking first abortion allows us to assess the situation of a woman requesting one abortion even though it may perform other abortions in the future.

## Conclusion

Health facilities providing abortion services need to pay more attention to women with repeat abortion. A first unplanned pregnancy may be an unfortunate accident for some women, but repeated ones cannot be regarded as an accident but a failure of the healthcare system in the education of women of childbearing age.

Results on CMD in women seeking abortions can be used to support interventions that might prevent mental health problems or help to treat its consequences to reduce the considerable burden of CMD experienced by these women. Further prospective studies are needed to well establish the relation between the number of abortion and the occurrence and the severity of CMD.
